# Lessons Learned for Online Health Community Moderator Roles: A Mixed-Methods Study of Moderators Resigning From WebMD Communities

**DOI:** 10.2196/jmir.6331

**Published:** 2016-09-08

**Authors:** Jina Huh, Rebecca Marmor, Xiaoqian Jiang

**Affiliations:** ^1^ University of California San Diego Department of Medicine La Jolla, CA United States

**Keywords:** qualitative research, online systems, social network, information science, Internet, social support, user computer interface, health information technologies, public health informatics, consumer health information

## Abstract

**Background:**

Online health community (OHC) moderators help facilitate conversations and provide information to members. However, the necessity of the moderator in helping members achieve goals by providing the support they need remains unclear, with some prior research suggesting that moderation is unnecessary or even harmful for close-knit OHCs. Similarly, members’ perceptions of moderator roles are underexplored. Starting January of 2013, WebMD moderators stopped working for WebMD communities. This event provided an opportunity for us to study the perceived role of moderators in OHCs.

**Objective:**

We examine the OHC members’ perception on OHC moderators by studying their reactions toward the departure of moderators in their communities. We also analyzed the relative posting activity on OHCs before and after the departure of moderators from the communities among all members and those who discussed moderators’ departures.

**Methods:**

We applied a mixed-methods approach to study the posts of all 55 moderated WebMD communities by querying the terms relating to discussions surrounding moderators’ disappearance from the WebMD community. We performed open and axial coding and affinity diagramming to thematically analyze patients’ reactions to the disappeared moderators. The number of posts and poster groups (members and moderators) were analyzed over time to understand posting patterns around moderators’ departure.

**Results:**

Of 821 posts retrieved under 95 threads, a total of 166 open codes were generated. The codes were then grouped into 2 main themes with 6 total subthemes. First, patients attempted to understand why moderators had left and what could be done to fill the void left by the missing moderators. During these discussions, the posts revealed that patients believed that moderators played critical roles in the communities by making the communities vibrant and healthy, finding solutions, and giving medical information. Some patients felt personally attached with moderators, expressing they would cease their community participation. On the other hand, patients also indicated that moderators were not useful or sometimes even harmful for peer interactions. The overall communities’ posting activity, which was already in decline, showed no significant difference before and after the moderators’ departure. In fact, the overall posting activities of the communities were declining well before the moderators’ departure. These declining posting activities might be the reason why WebMD removed the moderators.

**Conclusion:**

Compassionate moderators who provide medical expertise, control destructive member posts, and help answer questions can provide important support for patient engagement in OHCs. Moderators are in general received positively by community members and do not appear to interfere with peer interactions. Members are well aware of the possibility of misinformation spreading in OHCs. Further investigation into the attitudes of less vocal community members should be conducted.

## Introduction

In-person patient support groups, often organized by hospitals and clinical moderators, are a well-established mechanism to encourage peer-patient interaction, help patients improve self-efficacy, and educate patients about self-care management [1-4]. As Web 2.0 and social media spread as one of the main Internet activities, OHCs have also proliferated, often without moderators [5]. Unmoderated communities can suffer from the negative consequences of misinformation and poor social dynamics (eg, trolling) if not well-maintained by community members [6-8], especially when the interest of the community is health. The addition of moderators or active commitment by the members can diminish such negative consequences of OHCs [6-9]. However, the cost of resources is high for hiring moderators, preferably those with clinical backgrounds. In addition, moderating thousands of posts [5], and motivating moderators to voluntarily participate in OHCs can be difficult [10].

To successfully administer OHCs, we need to understand the critical role that moderators have in OHCs. A study revealed effective moderation styles for various negative online behaviors (eg, trolling) [11]. Although the effectiveness of moderation styles (eg, rewarding vs punishing) has been studied, there is no consensus regarding the necessity and role of moderators on OHC retention and improving levels of social support, where prior research reveals conflicting results. A study showed that moderators may be important for both the vibrancy of forums and improving patient outcomes [7,12]. Moderators review postings, redirect conversations, and stimulate dialogue when forum activity lags. They also execute a “process function” and help establish and enforce community rules [13]. Moderators offer valuable help that clinicians cannot provide, including suggesting ways to communicate with health care providers and finding useful health information resources for self-management [14]. The necessity of such external governance in moderating troll conversations may be dependent on the specific community. For those OHCs where patients have already established strong rapport with one another and are self-policing community conversations, external governance can be unnecessary [15] or sometimes even disruptive [16]. Online health communities independently run by patients only can self-maintain high information quality [17], although a systematic review showed that the effectiveness of purely peer-patient–based OHCs in terms of clinical outcomes lacks RCT-based evidence [18].

Unlike in face-to-face support groups, where moderators are often clinicians known to participants, moderators in OHCs do not have an immediate connection and the trust of members. Moderators are also unable to provide medical consultation. Furthermore, one prior study found that moderators urging patients to talk to their health care providers instead of consulting the community was highly associated with decreased peer-patient interaction [16]. Health experts might not understand the needs of patients, generating potential communication breakdown in OHC settings unless explicitly addressed [19]. Although young people who self-harm were willing to share their experiences with health professionals, the health professionals had low participation in an OHC [10]. The conflicting findings of prior research on patients’ expected moderator roles and utility of using moderators demonstrate the need for further investigation into the necessity of moderators in OHCs.

WebMD [20], an online health information portal, has run over 60 OHCs, in which staff moderators (STMs) and health professional moderators (HPMs) participated in peer-patient conversations as moderators. Staff moderators in WebMD were those without clinical backgrounds, who help facilitate and moderate discussions. For instance, they would ask questions such as, “What is your plan for this week? Please share with us” to facilitate conversations among the members and build rapport. As for responding to others’ threads, the moderators in WebMD were given guidelines as delineated in their policy statement [21], which says that the moderators are not supposed to provide medical consultations. Thus, STMs shared general information resources, such as pointers to accredited websites (eg, ADA.org).

Health professional moderators at WebMD were those with clinical backgrounds, providing clinical expertise to patient members by answering members’ questions and participating in conversations. Nevertheless, the HPMs would follow the same guidelines given to STMs, where HPMs would not be allowed to give personalized medical consultation. Health professional moderators could clarify clinical concepts and knowledge that would be useful for self-management, or direct ways to discuss with the members’ own health care providers.

WebMD, however, made a decision to let go of the STMs starting January of 2013. Members were not notified of this change in staffing prior to its occurrence, but quickly picked up on the absence of moderators. The departure of STMs triggered patient members to talk about their experiences with moderators, including HPMs, as part of the communities. The sudden departure of moderators from WebMD provided critical information about how patients perceived the benefits and disadvantages of having moderators in their communities when faced with loss of moderation.

While prior research has clearly demonstrated the benefit of moderators in OHCs, there is a limited understanding of the community member’s perceptions of moderators in their participation with OHCs. In this paper, we will investigate: (1) member’s perceptions of moderators in OHCs, and (2) the summary statistics analysis on OHC retention and moderators’ removal to understand both qualitative and quantitative aspects of moderators’ roles and influences in OHCs.

## Methods

We first walk through the data collection process, including community selection, followed by the descriptions on our mixed-methods analysis.

### Data Collection

Our institutional review board (IRB) determined this study to be unregulated because our data were publicly available and did not involve human subjects. WebMD has a total of 55 moderated communities. In January of 2015, we wrote an automated crawling program, which downloaded all 55 communities and saved the data to a local database software, MySQL (Oracle) [22]. The crawling program accessed the URLs of the WebMD OHCs, which are publicly available, and downloaded the content of the page (title, post content, date of post, and poster ID) in which OHC members exchanged discussions. The data in MySQL were then queried and analyzed for summary statistics analysis and exported to Microsoft Excel for thematic coding. Overall posting activity level of all 55 communities (Multimedia Appendix 1) was analyzed, which were the total number of posts, members, HPMs, and STMs to use the information for selecting the communities to study.

**Figure 1:**
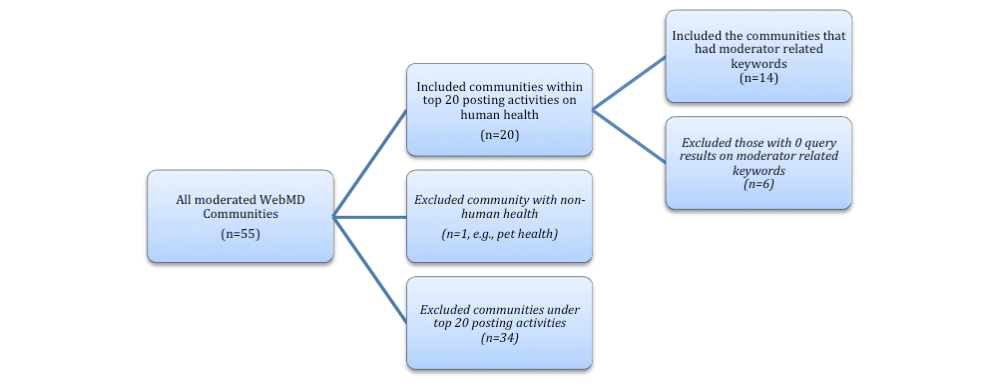
From all moderated WebMD communities (n=55), the communities were ranked based on total posting number. Then, top 20 communities with the most posting activities after excluding communities about nonhuman health-related topic (eg, pet health) were selected. Keyword search to find posts related to moderators was performed. All communities with more than zero query results when keyword search was performed (n=14) were included.

To best select active communities that can lend us more data regarding members’ discussions on moderators’ roles at WebMD, the following process was adopted: (1) From the total of 55 WebMD moderated communities, the top 20 communities which had the most posts in total after excluding nonhuman health-related community (eg, pet health) were selected. (2) Posts made before November 2012 and those made by moderators were excluded. (3) A regular expression query with the keywords related to moderators was conducted: “moderator[s],” “mod[s],” “[moderator names of that community].” The query was conducted on both the title and the message body of each post; (4) All posts under the threads that contained the query result posts were then collected. (5) The communities that came with zero query results when (3) was performed were excluded. (6) To further filter whether the thread was about (*a) moderators leaving* or (*b) members’ perceived moderators’ roles*, 2 coders manually went through each post to determine the relevance (further delineated in the Analysis section) and (7) The communities to investigate using our qualitative and quantitative analysis procedures were finalized. The result of this process is delineated in Figure 1 in the results section.

**Figure 2:**
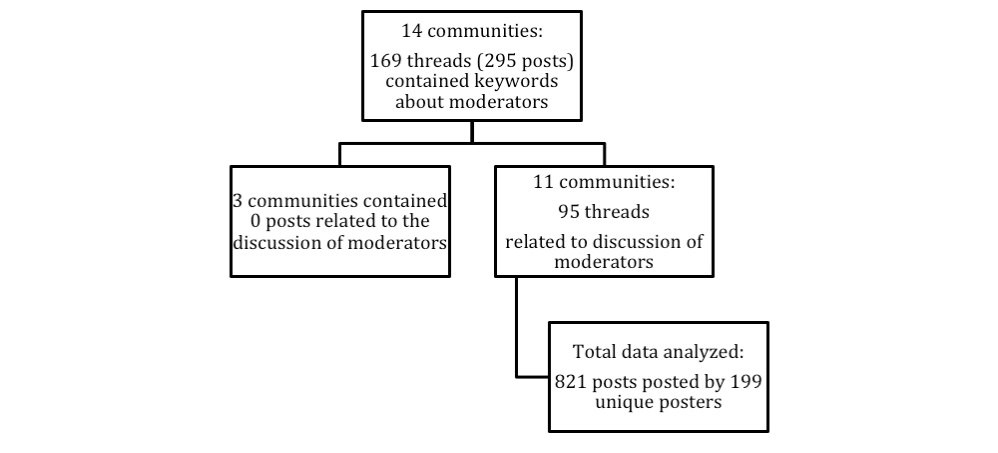
This figure describes further extracting related posts for this analysis from the 14 selected WebMD communities. From 14 selected communities, 11 communities contained related discussions of moderators. These posts were then tracked back to its original threads. All replies under those threads were retrieved, which came to be 821 total posts made by 199 unique posters.

### Analysis

We analyzed the total number of posts and unique posters grouped by STM, HPM, and members. Each poster group’s posting activities were recorded by month throughout the data collection period. Another study [14] showed that moderators triggered conversations among members by initiating threads, whereas replies were used to ensure members who asked questions were responded with welcoming responses. To understand such moderators’ member participation inducing effort together with members’ posting pattern, we also analyzed general posting activities such as making distinctions between replies versus thread initiating posts. The posting activities were visualized to investigate the overall posting pattern over time.

From the retrieved posts in step (6) of the data collection, 2 coders worked together with the first 5 communities on determining the criteria for relevance, asking about the posts that were related to discussion about moderators. If the post contained any dialogue about WebMD moderators—either STMs or HPMs, the post was flagged as relevant. The resulting criteria for relevance were as follows: First, the post should be about moderators, not other synonym that did not mean community moderators (eg, mod podge). Second, even though the post does not explicitly mention moderators’ removal from WebMD, if it contains any opinion about the moderators, it is included. The 2 coders divided the rest of the communities to code for relevance.

We analyzed the whole thread in which related posts were its part. One main reason for excluding the thread was that when the whole thread of a post ended without someone intervening to inform the poster that the moderator had left, in case a moderator left without the poster’s knowledge.

For the posts determined as relevant, a mixed-methods approach was adopted to understand the phenomenon using thematic analysis in addition to the quantitative methods employed. The thematic analysis [23] was used to identify topics and themes that emerged around discussions about the moderators’ disappearance or their perceived roles. These open codes were applied at the sentence level and were mutually inclusive.

With the resulting codes, an affinity diagram exercise was performed [24] (See Multimedia Appendix 2) to conduct axial coding. Affinity diagramming allowed us to find salient affinities among the codes. This process led to finding common and distinct themes across the codes, allowing main themes to emerge from the data. As a result, 2 main themes with three subthemes each were identified. These themes were applied at the post level, so each post would have multiple distinct themes, but a theme would not be repeated at the post level.

## Results

### Summary

In this section, we first report the community selection and the summary statistics of posting activities. We then further examine qualitative aspects of how members perceived having moderators, providing us with insights for how moderators should be implemented in OHCs. The follow-up posting activity analysis of the members who participated in the discussions was then reported.

### Community Selection

A total of 14 communities were found to have included moderator-related keywords after having filtered out nonhuman health-related communities and relatively less active communities.

### Filtering and Posting Activity Analysis

In this section, the results of the overall posting activities, identification of related posts for further qualitative analysis, and the posting activity analysis of the members who participated in the discussions about moderators leaving WebMD communities have been reported.

### Overall Posting Activities of Staff Moderators, Health Professional Moderators, and Members

For the 14 resulting WebMD communities for us to analyze, 6-15 STMs and 1-7 HPMs have been moderating each community. It was found that these communities had, on average, 650 members per STM (ranging from 260 members per moderator on the Breast Cancer community to 1526 members per moderator on the Sex and Relationships community). On average, STMs started 255 threads and posted 1115 replies (from 98 to 747 thread starting posts and from 102 replies to 3209 replies, n=14). On average, HPMs started 36 threads and posted 690 replies (from 0 to 119 thread starting posts and from 0 to 3348 replies). Further detailed breakdown of the activities are delineated in the Multimeda Appendix 1.

### Identifying Related Posts

Table 1 shows the query results and the filtering results on further identifying which posts were about moderators leaving the WebMD communities. From the 14 communities, a total of 293 posts under 169 threads were retrieved, which contained keywords about moderators. Of the 169 threads, 95 threads were related to the discussion about moderators’ exit. During this process, 3 communities were excluded from the analysis because all their query results were not found to be about the moderators’ exit. All replies from the 95 threads were then retrieved, which resulted in a total of 821 posts made by 199 unique posters under 95 threads by 11 communities.

**Table 1:** Eleven WebMD communities were identified as containing related posts on moderators leaving WebMD.

WebMD community	Number of posts identified as containing keywords (posts or number of threads the posts belong to)	Number of posts identified as related to moderators leaving (Number of total posts under the threads the related post belongs to)
Fibromyalgia	122 posts in 68 threads	78 posts from 33 threads (261 posts)
Breast_cancer	40 posts in 22 threads	31 posts from 14 threads (123 posts)
Diabetes	29 posts in 14 threads	23 posts from 9 threads (87 posts)
Back_pain	24 posts in 15 threads	20 posts from 11 threads (103 posts)
Pain_management	21 posts in 16 threads	12 posts from 9 threads (130 posts)
Sexual_conditions_and_sexually transmitted diseases	1 post in 1 thread	1 post from 1 thread (3 posts)
Sex_and_relationships	15 posts in 4 threads	15 posts from 4 threads (55 posts)
Bipolar_disorder	12 posts in 11 threads	9 posts from 8 threads (36 posts)
Lupus	12 posts in 3 threads	11 posts from 2 threads (13 posts)
Anxiety_and_panic_disorders	5 posts in 3 threads	1 post from 1 thread (2 posts total)
Depression	4 posts in 4 threads	3 posts from 3 threads (8 posts)
Diet	4 posts in 4 threads	0 threads (0 posts)
Pregnancy	3 posts in 3 threads	0 threads (0 posts)
Infertility_and_reproduction	1 post in 1 thread	0 threads (0 posts)
Total	293 posts in 169 threads	204 posts from 95 threads (821 posts)

### Activities of the 11 Communities

**Figure 3:**
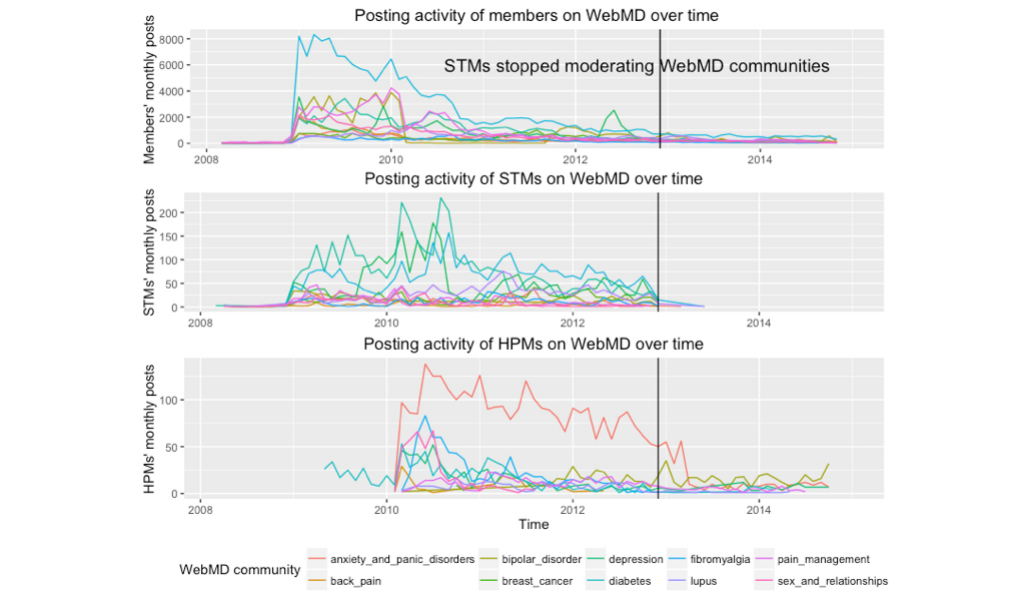
Posting activities of the members, STMs, and HPMs over time in the context of when STMs stopped moderating WebMD communities.

As shown in Figure 3, the overall posting activity of the members in 11 communities started declining during early 2010. This is 2 years before the STMs stopped moderating WebMD communities. For those communities with declining participation pattern, it can be seen that the moderators’ activities, both STM and HPM, have increased dramatically. However, members’ participation did not increase thereafter, and STMs’ activities have slowly decreased since late 2010 until STMs left WebMD communities. Staff moderators’ proportion of thread initiating posts ranged from 9% to 49% (Mean=26%). For HPMs, it ranged from 0% to 14% (Mean=6%). Detailed breakdown of this analysis is included in the Multimedia Appendix 1.

The last date of the STMs’ posts ranged between November 17 and December 20, 2012. Members’ posts related to moderators’ departure started as early as November 17, 2012 (the last posting date of the Sex and Relationships community) and persisted till December 20, 2012. There was a one-time post, an identical one, made by one STM in March to a few of the communities, asking members to share their experiences around insurance to a WebMD email address. Other than this particular post, no other posts have been made by STMs after December 20, 2012. One STM came back to the Fibromyalgia community with a regular member profile, updating about her current situation of finding a new job and health issues she is dealing with.

In December 2012 (when STMs last posted) and January 2013, the discussion about moderator leaving WebMD peaked. Since then, members continued to talk about how moderators left, sharing opinions and solutions around STMs’ departure.

### Members’ Qualitative Perceptions Toward Their Moderators

The thematic analysis of 821 posts resulted in generating 166 codes. From these codes, 6 subthemes grouped into 2 main themes from conversations about moderators during the period from December 2012 to February 2014 (see Table 2) were identified. All these coded posts were centered around moderators’ disappearance from the communities, which led to further discussions about members’ perceptions toward having moderators on the forum.

**Figure 4:**
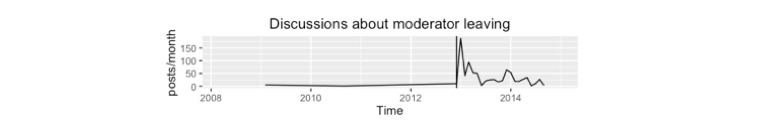
Members' discussion on moderator leaving peaked when moderators left and continued until the data collection ended. Some of the posts about moderator being gone were made as a reply to a post made a few years back (2009-2012). That is why earlier discussion points before December, 2012 are included in this graph.

The process by which the members figured out the news that the STMs have left WebMD was consistent across the 11 communities and is as follows: members first noticed moderators were gone; next, the groups shared the news individuals received from WebMD that the moderators had been removed from the forums; and finally, members pondered why the moderators had been removed, shared members’ initial reaction to the decision, and discussed how the community would continue to function in their absence (Figure 4).

**Table 2:** The table shows the number of coding instances and example quotes on WebMD users’ discussions around moderators’ departure.

Coding instances (frequency of code application) and example quotes	
Code name	Example quotes
**Figuring out that moderators are gone**	
Receiving the news	*I emailed about the absence of the moderators as well... I know many have been missing them and wondering where they went. This is the answer I got:* *WebMD has decided to shift the focus of the communities from being WebMD-managed to now being more member-managed, allowing members to shape their conversations and their communities in the direction that suits their needs. We have seen this as a growing trend among other social networking sites or message boards and feel it will better facilitate interaction among our members. Even with this change, we will continue to invite experts to keep answering member questions as they have been in the past. Along with this, we have also recently made some significant updates to our Answers tool (answers.webmd.com) and we hope you will make use of this resource as well. [Diabetes]* *I think it is a shame that WebMD did not communicate better with the members here [Fibromyalgia]*
Attempting to understand reasons for why moderators left	*I guess WebMD went through a change and decided that these people were no longer needed...I think though this is how companies do things like this these days. The less we know the better it goes is what they think, maybe. [Fibromyalgia]*
Discussing solutions to moderators’ departure	*We need to stay strong for each other and the new ones that come here for our help. [Breast Cancer]* *... As far as I know there aren’t any live moderators on but if there is a problem with a post, such as spam, etc, we all use the “Report This” link to bring it to one of the offline mods’ attention. [Depression]*
**Patient members’ experience with moderators**	
Moderators were not useful: Peer support is more important	*WebMD’s policy has always been to simply delete any thread that anyone ever complains about, which is ridiculous... I really can’t think of more than a handful of times over the years I’ve posted here that I saw a post including clearly inappropriate slang language. This is the one area in which the reduced moderator presence these boards is a feature, not a bug, IMHO. [Sex and Relationships] I have always maintained that the successful treatment of diabetes is 90% patient and only 10% doctor or caregiver [...] a lot of the ivory tower advice being given by those not affected by the disease may or may not apply to a particular patient. [Diabetes]*
Moderators provided pragmatic help	*Remember the good-ole-days when our Mods would assist and watch over us and delete such nonsense? [Breast Cancer]* *Some of you may recall I left the WebMD communities for several months earlier this year. I decided to come back and try again. Sadly, I can no longer continue supporting any organization or website that condones rude posts filled with vulgar language and free unethical advertising. While this does not happen often in the Back Pain Community, it is happening in other communities that I visit. Posts are reported but are not deleted in a timely fashion as they were when there were moderators in each forum. The attacks are getting more serious and are staying around for days before being deleted. I have decided to contact WebMD and have this account deleted. I can still read, but will no longer participate. I pray you all find effective ways to manage your back pain issues. Click on my username or avatar picture to read my story. Blessings - [Poster name anonymized] [Back pain]* *Why is no one removing the spam that is saturating this board????? [Bipolar Disorder]*
Established personal tie with moderators	*I just liked having her here and getting to know her and see her little ones pictures on here I will miss them also [Breast Cancer]* *Miss them all [Bipolar disorder]*

**Figure 5:**
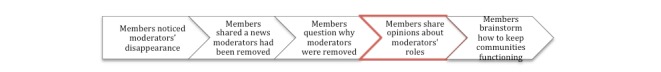
11 community members’ way to figure out moderators’ departure from WebMD. This process helped us understand members’ perceived role about moderators in online health communities.

### Figuring Out That Moderator has Gone

In figuring out moderators’ absence, members (1) noticed that moderators no longer participated, and (2) together they attempted to understand reasons for why moderators left. These discussions led to (3) discussions about solutions for not having moderators in their communities.

### Noticing That Moderators were Gone: Receiving the News

WebMD community members’ posts revealed that they had not received any public notification about STMs’ departure from the communities. Instead, members slowly started realizing in mid-January 2013 that their moderators were not posting on their communities any more. This finding demonstrates that moderators’ roles were salient enough that their absence was quickly detected by members. Members started noticing that moderators were no longer posting, and they were seeking reasons for the absence of moderators’ posts:

It seems that there are no more moderators on the community boards, and nobody is posting. Is anyone still here?

Lupus

Members who participated in multiple communities were able to publicize the idea that something suspicious was happening at the WebMD level, not just in one community:

I am active in four WebMD communities. No mods posting in any of them. Maybe they were given time off for the holidays?

Diabetes

To answer these questions, a number of members emailed the website directly and shared the replies from WebMD that WebMD had removed STMs from communities in December 2012 (see Table 1). Members who participated in multiple communities shared the news with other communities when suspicions from their members arose:

These communities no longer have moderators. The members are expected to inform others of the rules and policies. I am simply informing you of the facts.

Pain management

Members voiced their frustration about not being told about the change in the communities in advance:

NO GOODBYE? Just over. The top of the site still says moderator mediated. No one has said anything to us?

Fibromyalgia

As members realized moderators no longer participated in their communities, they attempted to figure out why moderators left.

### Attempting to Understand Reasons for Why Moderators Left

Members reacted strongly against moderators being removed from the communities and attempted to understand the rationale behind the decision. Members from the community suspected that budget cuts were the primary reasons for removal of the moderators. Members shared concerns about the moderators’ employment status, demonstrating the close relationships many had forged with the moderators. One member from a community shared evidence that WebMD had a budget cut, of which laying off moderators was a part. The following quote exemplifies the strong reaction to WebMD’s decision:

Why??? Why would they deliberately sabotage a great site like Web.M.D.??? For what purpose??? Where are the BIG CHIEFS ??? What have they to say for themselves???

Sex and Relationships

this is part of the changes which take place every day....businesses finding ways to cut or reduce costs....I am sure the moderators were paid for being here as are the other professional people who were here as well.

Fibromyalgia

In one community, a member posted a press release on WebMD’s budget cut, which they believed provided a reason for the layoffs:

*For all those who have made inquiries about our missing leaders, apparently this is the answer:* Forbes news release of December, 11, 2012 at 9:29AM

WebMD Cutting Staff 14%--­250 Jobs. In Cost Cutting Program WebMD this morning disclosed plans to cut 250 jobs about 14% of the company’s staff as part of a plan to reduce annual operating expenses by about $45 million. The company said the plan is designed to “streamline its operations, reduce costs, and better focus its resources on increasing user engagement, improving customer satisfaction, and driving innovation.” *(Breast Cancer)*

As members accepted that moderators were no longer with them and attempted to understand why such a decision was made, the discussion moved on to how they, as WebMD community members, should proceed in maintaining their communities.

### Discussing Solutions to Moderators’ Departure

Members discussed ways to independently manage their communities—to “stand united” (Breast Cancer, Diabetes, Sex and Relationships, and Pain management) and make sure members regulate and facilitate conversations themselves. Some members predicted lack of moderators would produce problems, and advocated for strong mechanisms for self-regulation; others believed that communities could thrive without moderators, so long as they could provide a strong support system for each other.

A few members stated that they no longer wanted to participate in the communities, as they were upset by WebMD’s decision to remove the moderators and bothered by increased spammers and trolls. One participant in the Pain Management community urged members to “ignore the trolls” and not “give [the trolls] any power to mess up” the community.

To sustain the communities, members needed to stay and support each other. For instance, members discussed “staying strong for each other and the new ones that come [to WebMD] for help” and urged that they “do [their] best to help [new comers] in any way [they] know how” (Breast Cancer). In response to some members stating they would leave, other members asked them to stay:

I’m sorry to see you go [David]. There are too few of us who have remained here over all the changes during the years (I’ve been around about 15 or more). Please reconsider. You help so many people. Anyway, hope you stay well and will contribute as you can to other groups.

Diabetes

I understand your needing a break, but you will be missed. Your insight and experience are a valuable asset to the other members of this community.

Pain management

Members also discussed that peer knowledge would not be enough and moderators were essential in communities requiring immediate answers to medical problems. Members discussed concrete actions they could take to fill the void left by the departure of the moderators, such as facilitating conversations by posting questions to elicit responses from “the wonderful people here” (Breast Cancer). After focused conversations about solutions to moderators being gone, members continued to encourage community members to monitor spam and advertisements. A member also shared one tactic of controlling malicious posts: “to not reply.” (Diabetes) 

*we can report it and see what happens and then FLAG IT AS SPAM by the FMILY here so no one bothers to read it! (Fibromyalgia)*

Members began to practice self-moderation on their communities. They would tell other members not to share medical advice and provided alternative ways to get help at low cost (Pain Management), thus mirroring the prior activities of paid moderators. Members also put self-moderation into practice by reporting people; this task was not favorably received and caused one member to note that she or he “really miss(es) the moderators” (Pain Management). Furthermore, members shared websites that members should be careful of visiting. Members encouraged those who stated they would leave the community to stay, in an effort to sustain the community.

### Members’ Experiences on Having Moderators

While making sense of moderators being gone, patients shared their past experiences of having moderators as part of their communities. Throughout this process, even though HPMs still stayed on WebMD, members discussed their perceptions about health professionals as moderators. Contrasting opinions emerged about HPMs. Patients had conflicting views of whether moderators gave pragmatic help. Patients expressed that moderators were critical in ensuring the community did not include hostile messages, spams, and misleading information. Patients reported they felt personally attached with moderators. On the other hand, some expressed moderators were not useful and that peer support was more critical.

### Moderators Provided Pragmatic Help

Of the members who commented on the utility of moderators, most mentioned moderators to be helpful. Although STMs were the ones who left the communities, members discussed experiences around HPMs as well, allowing us to understand members’ experiences around not just STMs but also HPMs:

I notice very few of the boards have doctors posting to them anymore. They were one of the reasons why I joined WebMD.

Sex and Relationships

Many members did not draw a clear distinction between HPMs and STMs until some members clarified that there is a difference between STMs and HPMs. This perception was best illustrated by the sentiment that medical expertise was a unique resource that moderators could provide the communities. For instance, members in Sex and Relationships discussed how they missed doctors on the forum and expressed “personal experience doesn’t always match having a medical opinion” and that although members “have tons of personal experience, [they] sure as heck don’t have the required learning *.* ” Similarly, a member stated, “While the support from people who are “suffering” from the same health problems is the most important thing on these blogs, expert advice [was] invaluable to [them] all. Members from the Diabetes community also felt that “the presence of mods experts in their fields to answer questions” was helpful. Members from Breast Cancer also noted the importance of moderators who are “(medical) experts” on the topic: and that they “had always awaited a promised ‘expert’ for this board” *(Breast Cancer).*

Although the STMs did not have the medical expertise, members appreciated the unique help provided by these STMs. These moderators helped keep the communities interested by posting articles, news, and information about WebMD. Members felt confident that moderators would find solutions to problems that arose on the forum. Members believed moderators ensured communities kept their “focus and no bickering.” Members did not “feel as safe posting here” (Diabetes, Back pain) as they did when the presence of moderators was obvious. Being monitored by moderators made some members feel safe to participate in the community. Members declared their intent to leave, saying that they “joined because it was not like all the other social networking sites" (Diabetes). They “did not feel comfortable here now” because “mods used to tend to these issues [monitoring spams] in a timely fashion and keep an eye out” (Breast Cancer). Apart from a policing role, moderators helped maintain communities by picking up conversation when it slowed down. Members felt people were not posting anymore because the moderators were gone.

Members from all communities saw an effect of not having moderators—spams lasting too long on the boards before being deleted, and that “New members are posting with rather radical ideas and trying to convince others that they are right” (Diabetes). The community being a “public, world-­wide site with the communities open to anyone” (Diabetes), not having moderation made many members no longer feel safe posting.

### Patients Felt Personally Attached With the Moderators

Members not only considered moderators to be helpful, but developed personal ties with them. They demonstrated these ties by voicing concerns about them losing their jobs. Several posters commented that moderators were like family members; for example, one wrote, “It is like losing a family member [...] To you all we will miss you” (Breast Cancer) and another similarly posted “we really loved having you on this board. We felt we were part of a large family” (Breast Cancer). In the Fibromyalgia community, members coordinated sending a card for one of the moderators. In the Depression community, a member started a thread to thank one of the moderators and wish her well. Some posters tried to petition WebMD to bring back the moderators, on the Sex and Relationships community, a thread called “BRING BACK OUR MODERATORS!!!” was posted with 17 replies in which the members vented on why moderators had to leave: “Complete madness to fire them.”

Several members even threatened to leave the communities after learning about STMs leaving WebMD. One member wrote how she had been on WebMD for over 10 years and said:

*It was and has been my life-­‐line and support for info from very good ladies. But Excuse ME-­‐-­‐-­‐You are NOT the only Medical Information site on the Internet. I do believe you have just Stepped in IT* [Breast Cancer].

Some members wondered where they could find their former community members on other websites. One member suggested to start a discussion titled ”Refugees from that other site“ for the members to meet up and continue to support one another (Breast Cancer). Another suggested Facebook site where members can find the other members. Several Breast Cancer community members discovered that former members had posted about a discussion thread called “Friends from WebMDs Support Board” at another breast cancer forum.

### Moderators Were Not Useful: Peer Support Is More Important

While most patients who commented about moderators believed they were helpful and important members of their communities, some patients discussed how they did not find moderators’ help to be useful. These members considered peer-support as the main benefit to participation in OHCs, and felt that moderators could deter the dynamic exchange of ideas among patients and peers.

For example, a member stated that HPMs did not weigh in with medical expertise when needed. Certain moderators’ questions were thought to be unhelpful for the community, such as “What turns you on?” in the case of Sex and Relationships community, which seemed to minimize the seriousness of the medical focus of the community. Other examples of ineffective moderation included inappropriate language spreading out even in the presence of moderators.

Members who saw moderators’ roles as providing medical expertise did not find HPMs to be helpful, as they cannot “treat, diagnose, or exam the people” (Sex and Relationships). In addition, members discussed how the “one size fit all” advice coming from the HPMs was not helpful; rather, peers’ “one-on-one, spiritually rewarding” support would be more beneficial for the community (Diabetes).

A few members considered others’ shared personal experience as more critical than moderators’ help. In an exchange between 2 members on the Sex and Relationships community, they shared their belief that their community did not seem to need HPMs’ involvement:

Member 1: It’s got to a point where we do not need a doctor, we’re too good Member 2: I think you are right? We really are better than any doctor, any day, Right???

A similar sentiment was found in the Fibromyalgia community:

*It’s the people that make the community and the support we gain from each other that keep us coming back.*

Some members believed that the absence of moderators might facilitate increased sharing among peers. Members believed that self-efficacy was essential and that moderators could not help with disease management. This thought was especially apparent on the Diabetes community. Patients felt that the communities did a good job of self-regulating and making sure that no one was claiming to share medical advice; rather, they were sharing anecdotal experiences. As one poster commented:

*I think there are a lot of very smart people who post on the various boards and I have never seen anyone claim to be an expert. We post what we know through personal experiences or something that has worked for us* [Sex and Relationships].

Although members believed that moderators may not improve the chemistry of communities, they did acknowledge that the website was losing long-term membership with the absence of moderators. Several members of the Diabetes community mentioned that it is up to the patients themselves to manage their disease, and that moderators cannot help. Similar sentiments were echoed on the Sex and Relationships community, noting that peer’s expertise was important.

## Discussion

### Principal Findings

OHCs were primarily developed to connect patients to others with similar disease processes in an attempt to provide emotional and informational support [25-28]. While some communities, such as WebMD, have experimented with incorporating paid moderators to guide discussions, it is generally believed that the primary utility of OHCs lies in peer-patient interactions [29,30] **.** Many believe that patients gravitate to these communities because they are free from professional governance [15,31]. By introducing and then removing STMs from the communities, WebMD has provided a critical test case to demonstrate the impact of moderators on OHCs.

This analysis of the departure of STMs from WebMD communities offers several insights into the role that moderators play on OHCs. Longstanding community members quickly detected moderators’ absence. The departure spurred conversation and debate on the communities about the utility of moderators. This rapid detection of the departure of moderators appeared to be indicative of the important role moderators played on many of the communities, such that the void was noted quickly. This void was validated by our quantitative analysis demonstrating that many of the moderators were active on communities. The close personal ties that many community members felt with moderators was demonstrated by the fact that many members worried about the employment status of the moderators, and some further petitioned WebMD to bring the moderators back.

Although many participants appeared to be especially appreciative of the pragmatic help that moderators had provided to their communities, such as monitoring for trolls and stimulating conversation when it had slowed, many of these roles were quickly assumed by community members. However, members did not enjoy these tasks, which often made them comment on how they missed the moderators. Community members also worked to create a sense of camaraderie and encourage their peers to continue to post, thus filling in the void left by the moderators. These tasks appeared to be less bothersome to posters. Such altruistic behavior is often observed in closely knit online communities, which is one of the strongest sources of maintaining online participation [32,33].

Several posters commented they did not feel as safe participating after moderators had left their communities, even as members tried to step in and report inappropriate posting activity. This finding suggests that even on vibrant communities, the role of the professional moderators may be unique and irreplaceable. This role of professional moderators, however, should be distinguished from general moderators or informal leaders in online community literature [34,35]. The sense of credibility and trust toward information source, including the author of the information, has a unique role in OHCs compared with general communities. The altruistic volunteerism common in most closely knit online communities [35-37] will not fulfill the absence of professional moderators in OHCs.

Although the general sentiment of community members toward moderators was positive, this sentiment was not universal. The departure of the moderators spurred debate on some communities, which felt that the utility of the moderators might vary according to the disease process the community dealt with. Members were insightful in their discussions about the tension between having moderators versus not having moderators. Having moderators can potentially impede conversation but limit the exchange of erroneous information. The lack of moderators or self-moderated communities can have strong community dynamics but requires strong commitment by the members to self-moderate bothersome, unmoderated exchanges by visitors and new comers with either ill intent or lack of knowledge around the community norms and common ground. Furthermore, not having moderators and committed members would lead to reduced access to health professional knowledge or validated information.

A few members threatened to, and eventually left their communities, after removal of the moderators. Some spoke about attempting to find people who had left for other websites. The remaining members believed that their communities were less active. However, this analysis has revealed that the overall posting activity of the WebMD health communities was declining throughout the study period, so it is difficult to infer that the decline in posting activity was a result of the moderators’ departure. Rather, the decline of member participation was followed by STMs’ and HPMs’ increased posting activity. Moderators’ increased activities showed WebMD’s effort to increase participation, which was evidenced by a study that reported qualitative analysis on the post content of WebMD’s moderators [14]. The researchers of this study found that moderators’ thread initiating posts were facilitating conversations through asking questions for members to share their experiences. Moderators’ replies were used to respond to threads to ensure members feel their posts were being read [38,39]. We saw that STMs generated more initiating of conversations compared with HPMs, and HPMs mainly replied to members’ posts, considering HPMs’ roles in WebMD was to provide clinical expertise [14].

The participation decline observed in WebMD is a canonical retention problem in social media. Thus, user migration has been widely studied to understand how to retain and attract users in social media platforms [40-42]. Main strategies in retaining users include providing useful content and improving socialization [42]. WebMD similarly made an attempt to spark conversations by adding moderators and their conversation facilitating messages together with HPMs’ contribution of clinical expertise. Even with increased moderators’ activities, however, members’ participation did not increase, which could have been a possible reason for WebMD’s decision to remove the moderators.

Another reason for decline in member participation might be due to the members not seeing health benefits as Su et al have discussed in their literature review of eHealth Systems [43]. However, the link between such individual evaluation of the community and the aggregate decline in participation is still unclear.

We also found the lack of distinction community members drew between HPMs and STMs. Although WebMD only dismissed STMs, members did not initially pick up on the fact that HPMs were still active members of their communities. Similarly, even on communities where there were HPMs participating (eg, Breast Cancer), some members were frustrated because they did not realize that there were HPMs (and such moderators were desired). Online health information consumers are keen on finding indicators for the source of information [44-46]. Thus, such WebMD members’ behavior to confuse moderators’ background expertise was a unique challenge worth noting. Our findings contest any perception that patients gravitate to OHCs because they are free from health professional involvement and that OHCs spread misinformation due to patients’ critical consumption of shared information online [47,48]. Many participants were concerned about the validity of information that would be shared on their communities without the expertise of paid moderators to help curate the information available to them. At the same time, assessing posters’ credibility and source of information was a challenging task, considering members’ observed confusion between STMs and HPMs.

Our study has implications for future OHC development. By exploring the impact of the removal of STMs from several well-established communities and the discussions ensued among participants, an insight was gained into what aspects of moderators’ presence and posting activity are most appreciated by members and helpful:

*OHCs need explicit, systematic policing activities put into place to help members feel safe and agree on continued participation.* This was a universal sentiment expressed on the communities; members appreciated this role that moderators had previously played and did not enjoy assuming this responsibility. Given the disproportionate number of verbal members who volunteer to take on the moderating role, strong dependence on these members will threaten the sustainability of the communities.

*The importance of HPMs may vary according to health topics of the communities*. It is difficult to characterize which communities benefited most from moderators from our study, given the more or less similar participation pattern across all communities. However, members’ conversations revealed the needs are different among different communities, where communities such as diabetes or heart disease might need immediate help from health professionals. Further work should clarify and identify what characteristics of illness experiences drive members to seek health professionals’ knowledge online rather than peers’ social support.

*The background of moderators (eg, clinical vs nonclinical) should be explicit*. Some WebMD community members demonstrated a poor understanding of the distinction between HPMs and STMs, where some thought HPMs have left the communities. Even in communities with an HPM, posters noted that they had been promised such an expert on the community and yet had still not encountered him. Explicit signatures should be available for every post and profile, clarifying if the moderator has undergone any professional health training, or is an STM. If an STM, whether the STM has any professional knowledge on health should be presented explicitly.

*Major community changes, such as removing moderators, should be communicated to members with advanced notice*. Much of the discussion about moderators centered around communities attempting to understand what had happened to them after they noticed their absence. Members’ frustration about the decision to remove moderators, and the lack of notification, was apparent in their posts. Members felt betrayed, and as many of them had developed close bonds with moderators, were upset about not being able to say their goodbyes to these members of their communities.

### Limitations

Our data source was limited to observed, publicly available posts. Thus, our analysis is limited to those who are verbal and have expressed opinions. Other rich metadata sources can be used to further analyze deeper insights about OHC activities, such as user logs, page views, and click streams [43]. The results reported in this study only account for a small percentage of the activities that can be observed in OHCs. However, access to such rich data on webpage activity of online health support group will require multiple layers of privacy protection methods, partnering with the support group provider, and close communication with the users who are willing to share such private data with the public for research purposes. Su et al [43] has also found that none of the eHealth systems studies on OHCs they reviewed examined private data, such as page views and other use logs. Studying such nonverbal behavior online will have continued challenges in obtaining the data and analyzing them.

### Conclusions

The analysis of STMs’ departure from WebMD communities provided a critical insight into the utility of moderators in OHCs. By analyzing the reactions of community members in 11 different illness communities to moderators’ departure, it was found that members perceive that moderators play an important role in OHCs, stimulating discussion and making them feel safe. However, this quantitative analysis of the posting activities shows that moderators’ efforts were not enough to increase the already decreasing member participation. Our work disputes a widely held belief about patients’ blindly accepting other patients’ shared information in OHCs and preferring nongovernance. Although moderators’ efforts did not play a large-scale influence on member participation, their presence was effective and favored by the core members. The discussion on member attrition and attraction in OHCs contributes to the call for analyzing attrition as a core research in eHealth systems [49]. This study contributes to the medical informatics community in understanding the utility of adding moderators and optimizing their roles in sustaining member participation in OHCs.

## Appendix

Multimedia Appendix 1 Table of posting activity of members, STMs, and HPMs in the WebMD communities.

Multimedia Appendix 2 Affinity diagram example of the codes generated.

## Abbreviations

STM: staff moderator
HPM: health professional moderator
OHCs: online health communities
RCT: randomized controlled trial
