# Supplementary material for: Lessons Learned for Online Health Community Moderator Roles: A Mixed-Methods Study of Moderators Resigning From WebMD Communities
Source: J Med Internet Res. 2016 Sep 8;18(9):e247. doi: 10.2196/jmir.6331 (PMC5034150; doi:10.2196/jmir.6331)
Supplement: Multimedia Appendix 1 [file jmir_v18i9e247_app1.pdf]

Appendix 1. Table of posting activity of members, STMs, and HPMs in the WebMD communities

	diabetes	pain_management	diet	sex_and_relationships	breast_cancer	fibromyalgia	pregnancy	back_pain	bipolar_disorder	sexual_conditions_and_stds	lupus	infertility_and_reproduction	anxiety_and_panic_disorders	depression
# of STMs	15	11	11	10	10	12	12	7	6	10	7	9	12	12
# of HPMs	3	3	6	3	1	5	2	6	1	0	2	8	2	4
# of members	5,571	7,752	5,696	15,275	2,613	9,344	8,679	3,992	2,615	1,1426	1,954	3,706	8,004	8,602
Total # of posts	71,073	36,539	15,972	73,789	25,889	172,632	21,934	30,458	60,491	41,680	15,009	56,442	24,449	54,064
Total # of threads	9,240	6,001	3,783	10,927	3,826	25,154	7,563	5,663	8,598	11,042	3,540	10,058	6,919	10,094
Total # posts by STMs	3,956	1,397	1,929	643	928	3,317	1,322	200	533	436	516	855	551	2,600
Averge # posted by STM	263.73	127	175.40	64.30	92.80	276.42	110.20	28.60	88.80	43.60	73.71	95.00	45.92	216.67
Min and max # posts by STM	1; 2,323	1; 1,363	2; 885	1; 253	1; 531	1; 3,217	1; 1,044	1; 156	1; 450	1; 236	1; 222	1; 602	1; 242	1; 1,303
Total # posts by HPMs	811	475	1,105	396	12	742	372	170	593	0	273	501	3,377	609
Averge # posted by HPM	202.75	158.3	184.2	132	12	148.4	186	28.3	593	0	136.5	62.63	1,688.5	152.25
Min and max # posts by HPM	19; 508	6; 287	1; 707	90; 215	12; 12	1; 680	1; 371	6; 52	593; 593	0	130; 143	1; 164	157; 3,220	9; 536
Earliest date of STM's post (m/d/y)	6/25/07	6/28/08	5/16/08	9/20/07	12/14/07	4/13/08	2/13/08	4/7/08	8/1/08	4/11/08	10/20/08	1/2/09	9/15/08	12/24/08
Latest date of STM's post (m/d/y)	12/10/12	6/13/13	12/10/12	11/17/12	12/10/12	6/13/13	12/10/12	12/5/12	12/3/12	10/18/12	12/10/12	12/3/12	3/27/13	12/10/12
Earliest date of HPM's post (m/d/y)	12/10/08	3/25/10	6/30/09	2/27/10	10/19/10	3/24/10	3/12/10	2/27/10	3/5/10	-	3/24/10	3/2/10	2/27/10	2/28/10
Latest date of HPM's post (m/d/y)	3/6/14	7/15/14	5/20/13	8/24/11	11/23/10	11/25/12	11/11/12	5/4/12	10/30/14	-	5/5/14	1/16/14	10/29/14	10/17/14
STM's # of thread initiating posts	747	187	438	305	245	294	209	98	204	121	195	160	144	226
STM's percentages of thread initiating posts	19%	13%	23%	47%	26%	9%	16%	49%	38%	28%	38%	19%	26%	9%
STM's # of replies	3,209	1210	1491	338	683	3023	1113	102	329	315	321	695	407	2374
HPM's # of thread initiating posts	82	21	119	47	-	55	1	2	1	-	20	68	29	27
HPM's # of replies	729	454	986	349	12	687	371	168	592	-	253	433	3348	582
