# Supplementary material for: Lessons Learned for Online Health Community Moderator Roles: A Mixed-Methods Study of Moderators Resigning From WebMD Communities
Source: J Med Internet Res. 2016 Sep 8;18(9):e247. doi: 10.2196/jmir.6331 (PMC5034150; doi:10.2196/jmir.6331)
Supplement: Multimedia Appendix 2 [file jmir_v18i9e247_app2.pdf]

Appendix 2. Affinity diagram example of the codes generated

			**Opinions around moderator being gone**										**Meta-community conversations for moderator being gone**	
				**Moderators manage community dynamic**			**Practical help by moderators**		**Deep engagement of mods**					
	**Moderator being gone does not matter if not better**						moderators were sincerely helpful		BRING THEM BACK; wish they would come back					
**Members did not notice moderators being gone**	**Moderators cannot help us**	**Unbounded moderation is harmful for community dynamic**		**Without moderators dynamics will be different**	**Moderators helped to make community vibrant**	**Moderators helped to moderate conversations**	**Moderators found solutions**	**Moderators give medical expertise**	**personally engaged with mods**	**WebMD is meaningless without moderators**		**Members attempt to understand reasons for moderators leaving**	**Members discuss becoming self-sufficient**	**Aftermath of Mods being gone**
realizing that moderators were gone took a while	moderators have not been useful	strict moderation and critique is harmful; GOOD no more strict moderation on languages; GOOD we can say what we want		PROB things are different now	PROB halts community vibracy	MODS helped to make WebMD a safe place	PROB mods helped find solutions	we want expert in this board	posts to the mods	mad at webmd		trying to understand why webmd fired moderators; trying to understand why moderators are gone; official reason for cutting staff	we can still be self-sufficient; we are self-sufficient	doctors don't post anymore either
user does not know	doctors cannot help us anyway	I like the chemistry without moderators		PROB questioning new people joining; worried about newbies	PROB people get no replies	PROB low quality of patient posts	PROB request to the moderator not accepted	lack of acknowledging the difference between HP and staff moderators	reminiscing the old times with the moderator	leaving webmd		discussing moderator cut as a sign for webmd cutting budget	we can be the moderators	WebMD has strengthened the Q&A tool instead
	users recognize the distinctive help that personal experience and medical expertise give separately	it wasn't that strict			MODS were the lifeline	PROB no moderation of spams	PROB cannot fix the forum	PROB who do we turn for medical expertise; doctors are good for medical expertise	the mods will be missed	staunch community member		it was a mistake to fire mods	we could be better in policing	cross community information flow
		moderators are more helpful when they engage in a different space				PROB patients have opinions but no supported facts		reasons for joining was because of doctor moderators	there are a few moderators that get talked about for each community			trying to figure out if they were fired	hard to define when to debunk patients sharing	different diseases have more urgency to have moderators
						arguing--one person does not have better knowledge than others; dont be critical of others			doctor moderator promotes her book				arguing--one person does not have better knowledge than others; dont be critical of others	
						flaming			moderator going was posted on a private board--core members knew about it					
									some moderators moved onto new jobs					
													unrelated	
